# Patient perceptions of dental students' professionalism in undergraduate prosthodontic clinics: a cross-sectional study

**DOI:** 10.3389/froh.2026.1802263

**Published:** 2026-04-24

**Authors:** Rasha A. Alamoush, Raneem Alkhader, Manar Mar’i, Suhad J. Al-Nasrawi, Marwa M. Alnsour, Julfikar Haider, Mahmoud K. AL-Omiri

**Affiliations:** 1 Department of Fixed and Removable Prosthodontics, School of Dentistry, The University of Jordan, Amman, Jordan.; 2 Department of Dentistry, The University of Jordan Hospital, Amman, Jordan; 3 Department of Conservative Dentistry, Faculty of Dentistry, University of Kufa, Najaf, Iraq; 4 Department of Restorative Dental Science, School of Dentistry, The University of Jordan, Amman, Jordan; 5 Department of Engineering, Manchester Metropolitan University, Manchester, United Kingdom

**Keywords:** dental professionalism, dental students, patient's perception, professional behavior, sociodemographic factors

## Abstract

**Purpose:**

This study evaluated the professionalism of dental students in dental clinics from the patients' perspective.

**Methods:**

A self-administered questionnaire was used to collect data from 312 patients attending undergraduate dental clinics at Jordan University Hospital where the invitation was extended to 350 participants and the response rate was 89.1%. The survey comprised four comprehensive sections: (1) sociodemographic participant information including age, gender, residency, education, and travel details, (2) dental student characteristics such as gender and academic year (4th or 5th year) (3) patients’ awareness of treatment, and (4) patients' satisfaction with the provided treatment. The data were analysed using SPSS. The Chi-square test was used to assess the significance of observed vs. expected item responses. Spearman's rho rank correlation analyzed associations between professionalism scores and participants' age, education, travel distance, and travel time.

**Results:**

The participants assigned high ratings (strongly agree and agree on responses) for the majority of the items. The median scores of most items were large (scores of 4 and 5) with a small interquartile range indicating participants' tendency to assign high scores for student professionalism in this investigation. Patients rated professionalism higher for short procedures (*P* = 0.046) and for female students (*P* = 0.046), regardless of the student's year of study (*P* = 0.840). The observed responses to the professionalism items were significantly different from the expected responses (*P* < 0.001). Older participants were assigned lower professionalism scores (Spearman's rho = −0.112, *P* = 0.048), while travel time showed no significant correlation with scores (*P* > 0.05). Higher education levels were associated with lower professionalism ratings (B = −1.106, *P* = 0.045, R² = 0.034), and female students received higher scores (B = 3.292, *P* = 0.039, R² = 0.014).

**Conclusions:**

Dental students demonstrated high level of professionalism, as reflected in patient ratings. Patients' perceptions were influenced by their sociodemographic factors and treatment duration, highlighting the importance of tailored approaches to clinical care.

## 1 Introduction

Professionalism describes the social contract between society and a given profession (1). In dentistry, professionalism has been defined as a set of competencies integrating values, behaviours, and attributes expected by both the profession and the public, based on ethical principles such as autonomy, beneficence, non-maleficence, and social justice (2). Professionalism as expressed from the patient side includes reliability, presented by timely access to competence; dignity, demonstrated by how the patient, staff and individual are being treated, the dedication to address the patient's needs and make them a priority and the quality of the clinical care the patients receive (3).

The General Dental Council of the United Kingdom has stated that professionalism is a core part of dental schools' curricula to ensure that the dental students acquire the technical knowledge and skills required to form professional attitudes, values, and ethical behaviour. According to the General Dental Council of the United Kingdom; the first aspect of professionalism is patients and the public which includes the following requirements: putting patients' interests first and acting to protect them, being honest and acting with integrity, respecting patients' dignity and choices, maintaining and protecting patients' information, recognizing and respecting the patient's perspective and expectations of dental care and the role of the dental team taking into account current equality and diversity legislation (4). Furthermore, the American Dental Education Association (ADEA) and the American Dental Association (ADA) recommend that all dental schools should have ethical and professional behaviour (5).

Professionalism is essential and hence it is a must to incorporate into the dental curriculum so that any deviation from the standards can be assessed and targeted. Hence, healthcare service quality is mostly evaluated through patient satisfaction (6). Research on dental professionalism from patients' perspectives is lacking, hence a greater focus on that aspect is highly recommended due to the importance of patients' satisfaction in the quality of dental services (7).

This study focuses mainly on fixed and removable prosthodontics as they are considered the core of dentistry and have essential educational and clinical implications. Fixed prosthodontics involves the placement of non-removable restorations, such as crowns and bridges, which are directly attached to existing teeth or implants. It requires a deep understanding of tooth preparation, material selection, and occlusal principles to ensure functional and aesthetic outcomes for patients. Hence, it reflects students' precision in technique, patient management, and subsequently their professional level. Similarly, removable prosthodontics is equally vital as it involves providing removable prostheses for complete or partial edentulous patients. It requires students to manage various clinical scenarios, including patient comfort, retention issues, and adjustments over time. Therefore, fixed and removable prosthodontics is the core of a comprehensive educational experience that prepares students to meet diverse patient needs effectively while fostering professionalism in their practice (8).

Professionalism in dental education includes communication skills, emotional intelligence, and patient-centred care. Effective dentist–patient communication and emotional intelligence significantly influences patient satisfaction, and treatment satisfaction (9, 10). Further, structured communication training, educational strategies such as interactive learning and video-based training also enhance students' ability to engage with patients and improve their understanding of professional behavior demonstrate empathy, and effectively discuss treatment plans (11–13).

Previous studies have explored professionalism in many healthcare fields, including dentistry (7, 14), Research in dental education settings has predominantly focused on self-assessments by students (6), evaluations by faculty (15) and general patients' viewpoints (16). Several studies assess patient perspectives—especially in the context of communication skills (17, 18). However, there is a lack of studies evaluating patient-based perceptions of dental student professionalism across different clinical procedures, particularly concerning long- and short-term clinical procedures. Therefore, it is clear that current research is limited in assessing dental professionalism from patients' perspectives, with a specific focus on core dental specialities: Conservative dentistry and prosthodontics. Moreover, there is limited evidence on sociodemographic factors influence patient expectations and evaluations of professionalism, especially in Middle Eastern countries. Sociodemographic factors such as age, gender, education level, and travel-related variables may influence patient perceptions of professionalism. These factors might shape patients' expectations, communication preferences, and previous healthcare experiences, which affect how professional behaviour is interpreted and evaluated. Therefore, examining such variables is crucial to understand variability in patient assessments and subsequently developing patient-centred approaches to professionalism in dental education (15).

This study aims to evaluate dental students' professionalism from the patients' perspective in the Fixed and Removable Prosthodontics Department at the School of Dentistry, The University of Jordan. The study objectives are to: I) Assess the level of dental student professionalism as perceived by patients. II) Compare professionalism scores across student gender and academic year. III) Examine differences in professionalism between short-term and long-term procedures. IV) Investigate the influence of sociodemographic factors (age, gender, education level, and travel-related variables) on patient evaluations of professionalism.

## 2 Subjects and methods

### 2.1 Ethics approval

The research protocol was approved by the Ethical Committee of the Faculty of Dentistry of the University of Jordan (Reference: 30–2023) and was in full accordance with the world medical declaration of Helsinki. All the participant patients were informed regarding the aim and objectives of the questionnaire and agreed to fill out the form voluntarily. No personal information of patients or students were exposed or traced back to individuals as the questionnaire was anonymized and an appropriate protocol was followed so that personal information were securely stored.

### 2.2 Study group and design

The study was cross-sectional and was conducted in the students' dental clinics Fixed and Removable Prosthodontics departments in the academic year 2022–2023. The study group included all students' patients who attended dental clinics and consented to participate. Each student was evaluated by one patient only.

Data were collected using a self-administered questionnaire that investigated the professionalism from the patient's perspectives at undergraduate dental clinics at Jordan University Hospital at the end of their treatment procedure. Patients completed the questionnaire immediately after completing long term treatment (more than 3 visits) or short term treatment (1 visit). The questionnaire was adopted and adjusted from previous publications where items number 2, 9–12, 17–26, 28, and 30 in Supplementary Appendix 1 are from reference number (16, 19). and items number 1, 3–8, 13–16, and 27 are from reference number (11). The study has four sections. The first section includes sociodemographic information of the participants: age (stratified into 4 age groups: 20–39 years, 40–59, 60–79, and 80–100), gender, residency (rural or urban), education (stratified into no education, primary, middle or high school, diploma, bachelor, and master's degree), distance travelled to reach the hospital, and travel time required to reach the hospital.

The second section includes the physical attributes and behaviour of dental students and their information including gender and academic year. The third section covers patients' awareness of the treatment provided and the last section contains patients' satisfaction with the provided treatment. Any questionnaire that had missing information was excluded. Patients were asked specific questions regarding the behaviour, communication and appearance of the students. The questionnaire was scored based on the participants' answers according to an agreement scale (Likert scale), from strongly disagreed (score of 1) to strongly agreed (score of 5). A total professionalism score was obtained from each participant by adding the sum of the scores of the 32 items in the third and final sections of the survey (Supplementary Appendix 1), with a possible minimal score of 32 and a possible maximum score of 160.

### 2.3 Inclusion and exclusion criteria

The inclusion criteria were as follows: Students' patients who attended dental clinics at Jordan University Hospital and patients who consented to participate. Patients who attended the Fixed and Removable Prosthodontics departments and patients who visited the clinics in the academic year 2022–2023. The exclusion criteria were as follows: questionnaires with missing information were excluded and patients who visited clinics for check-ups only and patients who received no treatment.

### 2.4 Sample size calculation

The G*Power computer software (version 3.1.9.7; Heinrich-Heine University) was used to estimate the sample size for this investigation. It should be noted that a non-probability convenience sampling method was employed for sample size estimation to accomplish meaningful statistical comparisons. Although G*Power is typically used with probability sampling, it has been applied in non-probability contexts to ensure adequate power when statistical comparisons are made. Nonetheless, this approach is acknowledged as a limitation of our sampling strategy.

An estimated total sample size of 234 participants was identified following an input of two-tailed *α* probability error of 0.05, an effect size of.3, and a study power (1—*β*) of 0.8 in *a priori* power analysis using a *t*-test family for means with the Mann–Whitney test. Further participants were included in this investigation to secure the required estimated sample size. The invitation to participate in this investigation was extended to 350 participants, and 312 participants responded (89.1% response rate). None of the participants were excluded after being included in the study.

All patients (350 patients) who attended the students' dental clinics during the time of the study were invited to participate.

### 2.5 Study validation

The authors developed a rigorous survey validation process involving comprehensive translation and expert review. In addition, the survey development and study validation steps included incorporating expert panel feedback, obtaining panel approval, and preparing the survey for the pilot study. Furthermore, the survey items were adopted from the literature review which has added to the validity of the study (7, 16, 19).

After preparing and selecting the questions, the questionnaire was meticulously translated from English to Arabic using forward and back-translation techniques, with bilingual experts ensuring linguistic accuracy and meaning preservation. The translation process carefully considered potential linguistic nuances to ensure patient comprehension, particularly for Arabic-speaking participants. Then, the initial draft of the survey was prepared by the authors after being reviewed to avoid duplication or repetition of the items.

Then, the survey draft was reviewed by a panel of three academic professionals with a dental background (university professors from two dental schools in Jordan and Iraq) who had more than 15 years of experience in university and dental education and had long experience in questionnaire and survey-based studies. The panel was requested to provide critical insights into survey and question clarity and relevance, and to provide any further comments or suggestions regarding the survey including suggestions for adding new items, removal of items, and need for item modification, revision or adjustments. The panel was requested to provide a feedback on the clarity of each question and whether it was easy to understand by choosing one of the following responses: not clear, need significant change/revision to be clear, need minor change/revision to be clear, and very clear. The panel was also requested to provide a feedback on the relevance of each question to the intended measurement of professionalism of students as rated by patients by choosing one of the following responses: not relevant, somewhat relevant, quite relevant, and highly relevant. All the items were judged to be highly relevant by the expert panel. Meanwhile, only two items (items 31 and 32) were judged to need minor adjustments for clarity.

After receiving the panel review, the content validity index for the relevance of each item (item content validity index) was calculated by dividing the number of agreements between experts on the relevance of the item by the number of experts; and was found to be 1 for each item (Supplementary Appendix 1). Also, the content validity index for the relevance of the survey (scale content validity index) was 1 (Supplementary Appendix 1). Similarly, the content validity index for the clarity of each item (item content validity index) was calculated by dividing the number of agreements between experts on the clarity of the item by the number of experts; and was found to be 1 for each item (Supplementary Appendix 1). Also, the content validity index for the clarity of the survey (scale content validity index) was 1 (Supplementary Appendix 1). Consequently, the content validity index values indicated that the survey had adequate validity.

Then, the survey was prepared accordingly to be tested in a pilot study. For this purpose, a pilot study with a small sample group of interns (12 participants) was conducted to test for comprehension of questions, clarity of Likert scale responses, potential misinterpretations, and reliability. The participants in the pilot study did not report any difficulty in understanding and answering the questions of the survey. The pilot study results demonstrated that the Cronbach's Alpha value for the survey was 0.902 (scale mean = 146.6, variance 166.08, SD 12.89, number of items = 32); indicating proper internal consistency and reliability of the survey.

The above described methodical approach ensured a high-quality, linguistically appropriate research tool that maintained the original research intentions while being accessible to the target population. After that, the survey was finalized and prepared for the main data collection. The main study results demonstrated that the Cronbach's Alpha value for the survey was 0.885 (scale mean = 140.6, variance 186.58, SD = 13.66, number of items = 32); indicating proper internal consistency and reliability of the survey. Furthermore, a confirmatory factor analysis indicated that the data adequately fitted the structure of the survey as the eigenvalue of item loading onto the total professionalism score was >0.4 for each item (Supplementary Appendix 2).

### 2.6 Statistical analysis

The statistical analysis for this investigation was conducted using the SPSS computer software (IBM SPSS Statistics v20.0; IBM Corp., USA). Regarding data distribution, the variables were either non-normally distributed (total professionalism scores: Shapiro–Wilk test statistic = 0.941, *P* < 0.001) or categorical. Following normality testing of the data distribution, the variables were either non-normally distributed (total professionalism scores) or categorical. The median, interquartile range, and range were used to describe the non-normally distributed continuous and ordinal variables, while percentages and frequencies were used to describe other categorical variables.

Since the data was not normally distributed or has ordinal nature of Likert scale, the non-parametric tests were used for group comparisons (Mann Whitney *U*-test) and identification of correlations (Spearman's Rho rank correlations). The levels of student professionalism were compared between 4th and 5th year students, between males and females, and between long (prosthodontic) and short (conservative) dental procedures using the Mann Whitney *U*-test. Effect size for group comparisons using Mann Whitney *U*-test was calculated using r statistic. Raw correlations between individual item scores and each of the participants' age, education, distance of travel to reach the hospital, and the time of travel to reach the hospital were calculated using the Spearman's Rho rank correlations.

The predictors of professionalism scores were tested using multiple linear regression analysis (stepwise and hierarchical) while controlling for demographic confounding variables including age, education, travel time, and travel distance. Although the total professionalism scores were not normally distributed, the multiple linear regression was appropriate for use with the total professionalism scores in this study because the values of Kurtosis (value equals 1.525 with a standard error of 0.275) and skewness (value equals −1.018 with a standard error of 0.138) of the standardized and unstandardized residuals of the total professionalism score were within the acceptable ranges for a normal distribution and met the assumptions for using multiple linear regression. In addition, the normal P-P plots showed linear trend between the standardized residual values and predicted values of total professionalism scores. This met the linearity assumption of linear regression analysis. Also, the normality of the residuals was verified via the scatter plots (Standardized residuals were scattered between −3 and 3 values without presence of trends of values within the scatter). This also showed constant variance (Homoscedasticity) across the levels of the total professionalism score. Moreover, Durbin-Watson test values (1.964 and 1.969) indicated that no autocorrelation (Independence of observation) assumption of linear regression was met. Finally, no multicollinearity was found as indicated by the values of tolerance and variance inflation factor (VIF). A two-tailed *α* of 0.05% and 95% confidence intervals were considered for statistical significance during this investigation.

## 3 Results

A total of 312 participants were recruited for this study, and their data was processed and analysed. Table 1 presents the distribution of the demographic variables among the study sample including participants' age, level of education, occupation, distance travelled to reach the hospital, and time of travel to reach the hospital. The participants who received long treatment had lower levels of education than those who received short treatment (Mann Whitney *U*-statistic = 8,816.5, *Z* = −4.301, *P* < 0.001, Effect size *r* = 0.060)). In addition, the participants who received long treatment were of older age categories (*P* < 0.001, Effect size *r* = 0.524)) and travelled longer distances to reach the hospital (*P* = 0.008, *r* = 0.022) than those who received conservative treatment (Table 2).

**Table 1:** Distribution of demographic variables and variables regarding information about treatment and students who provided the treatment among the study population (*n* = 312).

Variables		All Sample (*n* = 312)		Prosthodontics (*n* = 159)		Conservative (*n* = 153)	
		*n*	%	*n*	%	*n*	%
Age	20–40 years	121	38.8	9	5.7	112	73.2
	40–60 years	101	32.4	65	40.9	36	23.5
	60–80 years	86	27.6	81	50.9	5	3.3
	80–100 years	4	1.3	4	2.5	0	0
Education	None	21	6.7	11	6.9	10	6.5
	Primary school	28	9.0	26	16.4	2	1.3
	Secondary school	61	19.6	39	24.5	22	14.4
	High School	94	30.1	40	25.2	54	35.3
	Diploma	47	15.1	20	12.6	27	17.6
	Bachelor	58	18.6	21	13.2	37	24.2
	MSc/PhD	3	1.0	2	1.3	1	0.7
Occupation	Unemployed	130	41.6	83	52.2	47	30.8
	House wife	72	23.1	21	13.2	51	33.3
	Employee	105	33.7	52	32.7	53	34.6
	Military	5	1.6	3	1.9	2	1.3
Traveled distance	Less than 10 km	67	21.5	29	18.2	38	24.8
	10–30 km	131	42.0	60	37.7	71	46.4
	More than 30 km	114	36.5	70	44.0	44	28.8
Travel time	15 min	49	15.7	27	17.0	22	14.4
	30 min	83	26.6	36	22.6	47	30.7
	60 min	133	42.6	65	40.9	68	44.4
	120 min	47	15.1	31	19.5	16	10.5
First time to be treated by students	No	76	24.4	40	25.2	36	23.5
	Yes	236	75.6	119	74.8	117	76.5
Level of dental student you visit	4th year	163	52.2	81	50.9	82	53.6
	5th year	149	47.8	78	49.1	71	46.4
Gender of dental student you visit	Male	118	37.8	61	38.4	57	37.3
	Female	194	62.2	98	61.6	96	62.7
Would be treated by students if you have insurance	No	42	13.5	31	19.5	11	7.2
	Yes	270	86.5	128	80.5	142	92.8
How did you know about treatment in the hospital	Social media	130	41.7	61	38.4	69	45.1
	Dental students	30	9.6	10	6.3	20	13.1
	Others	152	48.7	88	55.3	64	41.8

**Table 2:** Differences in age, travel distance, and travel time between participants who received long (prosthodontics) and short (conservative) procedures among the study population (*n* = 312).

Variables	Statistic	All Sample (*n* = 312)	Prosthodontics (*n* = 159)	Conservative (*n* = 153)	MWU	*Z*	*P*	ES
Age	Median	2	3	1	2,570.5	−12.788	<0.001	0.524
	IQR	2	1	1				
	Range	1–4	1–4	1–3				
Travel distance	Median	2	2	2	10,181.5	−2.671	0.008	0.022
	IQR	3	1	2				
	Range	1–3	1–3	1–3				
Travel time	Median	3	3	3	11,202.0	−1.275	0.202	0.006
	IQR	1	1	1				
	Range	1–4	1–4	1–4				

IQR, Interquartile range; MWU, Mann Whitney *U*-test statistic; Z, Z statistic; P, Probability value; ES, Effect size using r statistic.

Table 3 shows the distribution of participants' responses regarding dental students' professionalism among the total study population. Most of the responses to individual items had a large median (score of 5) with a small interquartile range indicating that the participants tended to assign high scores for student professionalism in this investigation. Also, the sum of all individual item scores (total professionalism score) was high (median = 144, interquartile range = 20), and ranged between 85 and 160 across the total study population (Table 3). The frequencies and percentages of the responses of participants to individual items are presented as supplementary material to this manuscript (Supplementary Appendix 3).

**Table 3:** Distribution of participants’ responses regarding dental students’ professionalism among the study population (*n* = 312).

Questions	Total sample (*n* = 312)			Prosthodontics (*n* = 159)			Conservative (*n* = 153)		
	M	IQR	Range	M	IQR	Range	M	IQR	Range
1. First impression affected confidence in student's abilities.	5	1	1–5	5	1	1–5	5	1	1–5
2. Reception at the clinic influenced confidence.	5	1	1–5	5	1	1–5	5	1	1–5
3. Student's attire inspired assurance.	5	1	1–5	5	0	2–5	5	1	1–5
4. Physical appearance/attire affected perception of future care.	5	1	1–5	5	1	1–5	5	1	1–5
5. Appearance and behavior exemplified professional integrity.	5	0	1–5	5	0	3–5	5	1	1–5
6. Should wear a white lab coat rather than a surgical smock or scrubs.	3	1	1–5	3	2	1–5	3	1	1–5
7. Appearance/attire affected comfort level.	4	2	1–5	3	2	2–5	5	1	1–5
8. Appearance/attire affected anxiety level.	3	2	1–5	3	1	1–5	3	3	1–5
9. Effective time management showed competence.	5	1	1–5	5	0	2–5	5	1	1–5
10. Overall conduct conveyed competence and increased comfort.	5	0	2–5	5	0	2–5	5	1	2–5
11. Student's behavior improved perception of dentists.	5	1	2–5	5	0	2–5	5	1	2–5
12. Maintained infection control and orderly environment.	5	0	2–5	5	0	3–5	5	1	2–5
13. Displayed professionalism comparable to faculty.	4	2	1–5	3	4	1–5	4	1	2–5
14. Seemed well prepared for the procedure.	5	0	1–5	5	0	2–5	5	1	1–5
15. Demonstrated knowledge to resolve my problems.	5	1	2–5	5	0	2–5	5	1	2–5
16. Trusted student to provide the best care.	5	0	1–5	5	0	1–5	5	1	2–5
17. Chief complaint was fully heard.	5	0	2–5	5	0	2–5	5	0	2–5
18. Explained what needed to be done and why.	5	0	1–5	5	0	1–5	5	1	3–5
19. Informed about diagnosis and treatment options.	5	0	1–5	5	0	1–5	5	1	2–5
20. Informed about treatment duration beforehand.	5	1	1–5	5	0	1–5	5	1	2–5
21. Informed about risks and benefits of treatment.	5	1	1–5	5	3	1–5	5	1	1–5
22. Explained retention and stability of the prosthesis.	4.5	1	1–5	4	3	1–5	5	1	1–5
23. Explained expected aesthetics of the prosthesis.	5	1	1–5	4	2	1–5	5	1	1–5
24. Explained expected disadvantages of the prosthesis.	4	2	1–5	4	3	1–5	5	1	1–5
25. Explained effects on tissues and virtual age due to prosthesis.	4	2	1–5	4	3	1–5	4	2	1–5
26. Used appropriate language and explained technical terms.	5	0	1–5	5	0	2–5	5	1	1–5
27. Encouraged and clearly answered questions.	5	0	2–5	5	0	4–5	5	1	2–5
28. Clarified assumptions with clear facts.	5	0	1–5	5	0	3–5	5	1	1–5
29. Respected cultural and religious sentiments.	5	0	3–5	5	0	4–5	5	0	3–5
30. Informed about rights in accepting/rejecting treatment plan.	5	0	1–5	5	0	2–5	5	1	1–5
31. Satisfaction with overall treatment experience at JUH.	5	1	2–5	5	1	2–5	5	1	2–5
32. Likelihood of recommending the student to others	5	0	2–5	5	0	3–5	5	0	2–5
Total professionalism scores	144	20	85–160	143	16	112–160	145	25	85–160

Prosthodontic, Participants received long prosthodontic procedures; JUH, Jordan University Hospital; Conservative, Participants received short conservative dental procedures; M, Median; IQR, Interquartile range.

Table 4 demonstrates the variations in participants' responses regarding dental students' professionalism based on the type of dental procedure, the gender of the students, and the student's year of study. The total professionalism scores were higher when participants were receiving conservative short procedures (*P* = 0.046, Effect size *r* = 0.012) and when they were treated by female dental students (*P* = 0.046, *r* = 0.012); regardless of the year of study of the dental student (*P* = 0.840, *r* = 0). However, 4th year students received higher professionalism ratings than the 5th year students did considering some individual items including items number 3 (students ability ensured by their dress), 4 (student's appearance affected participants' feelings about care), 16 (student took best care of participants), and 17 (participant's main complaint was heard fully and in detail) (*P* < 0.05, Table 4). In addition, female students received higher professionalism ratings than the male students did regarding items number 2 (reception in the clinic affected participants' confidence) and 10 (student conduct conveyed competence and increased participants' comfort) (*P* < 0.05, Table 4). Furthermore, the participants who received long treatment assigned higher professionalism scores for the students regarding items number 3, 5, 9–12, 14–18, 20, 26–30, and 32 (*P* < 0.05, Table 4). Meanwhile, the participants who received conservative treatment assigned higher professionalism scores for the students regarding items number 7, 8, 13, and 21–25 (*P* < 0.05, Table 4).

**Table 4:** Variations in participants’ responses regarding the professionalism of dental students based on the type of procedure received by the patients, gender of dental students, and the study year of the dental students (*n* = 312).

Questions	Type of procedure				Students’ gender				Students’ study year			
	MWU	*Z*	*P*	ES	MWU	*Z*	*P*	ES	MWU	*Z*	*P*	ES
1. First impression affected confidence in student's abilities.	11,199.5	−1.339	.181	0.006	10,502.0	−1.351	.177	0.006	11,127.5	−1.315	.188	0.006
2. Reception at the clinic influenced confidence.	11,903.0	−.392	.695	0.000	9,707.0	−2.699	.007	0.024	11,566.0	−.763	.446	0.002
3. Student's attire inspired assurance.	9,400.0	−4.313	<.001	0.060	11,226.5	−.353	.724	0.000	10,291.5	−2.796	.005	0.026
4. Physical appearance/attire affected perception of future care.	11,526.5	−.899	.369	0.002	11,120.5	−.474	.636	0.000	10,089.0	−2.813	.005	0.026
5. Appearance and behavior exemplified professional integrity.	8,558.5	−6.168	<.001	0.122	11,074.0	−.656	.512	0.002	11,054.0	−1.753	.080	0.010
6. Should wear a white lab coat rather than a surgical smock or scrubs.	10,805.5	−1.781	.075	0.010	11,425.0	−.028	.977	0.000	11,916.5	−.201	.841	0.000
7. Appearance/attire affected comfort level.	8,871.5	−4.433	<.001	0.062	11,048.5	−.552	.581	0.000	11,871.0	−.268	.789	0.000
8. Appearance/attire affected anxiety level.	10,454.0	−2.222	.026	0.016	10,457.0	−1.325	.185	0.006	10,754.5	−1.717	.086	0.010
9. Effective time management showed competence.	9,315.5	−4.335	<.001	0.060	10,551.5	−1.404	.160	0.006	11,895.0	−.267	.790	0.000
10. Overall conduct conveyed competence and increased comfort.	8,681.0	−5.860	<.001	0.110	10,093.5	−2.346	.019	0.018	12,054.5	−.025	.980	0.000
11. Student's behavior improved perception of dentists.	9,291.0	−4.516	<.001	0.066	10,351.0	−1.775	.076	0.010	11,346.5	−1.145	.252	0.004
12. Maintained infection control and orderly environment.	8,963.0	−5.943	<.001	0.114	10,452.5	−1.902	.057	0.012	11,500.5	−1.067	.286	0.004
13. Displayed professionalism comparable to faculty.	7,296.0	−6.391	<.001	0.130	10,431.0	−1.374	.169	0.006	10,688.0	−1.823	.068	0.010
14. Seemed well prepared for the procedure.	7,963.5	−7.150	<.001	0.164	10,399.5	−1.837	.066	0.010	11,384.5	−1.176	.240	0.004
15. Demonstrated knowledge to resolve my problems.	8,938.5	−5.012	<.001	0.080	10,669.0	−1.245	.213	0.004	11,687.0	−.598	.550	0.002
16. Trusted student to provide the best care.	9,778.0	−4.189	<.001	0.056	10,436.0	−1.828	.068	0.010	10,809.0	−2.234	.025	0.016
17. Chief complaint was fully heard.	9,540.0	−5.622	<.001	0.102	10,885.0	−1.239	.215	0.004	11,011.5	−2.300	.021	0.016
18. Explained what needed to be done and why.	9,533.5	−4.827	<.001	0.074	10,580.0	−1.638	.101	0.008	11,495.5	−1.064	.287	0.004
19. Informed about diagnosis and treatment options.	11,185.0	−1.640	.101	0.008	10,474.5	−1.678	.093	0.010	11,695.0	−.630	.529	0.002
20. Informed about treatment duration beforehand.	9,348.0	−4.658	<.001	0.070	10,473.0	−1.659	.097	0.008	12,020.5	−.081	.935	0.000
21. Informed about risks and benefits of treatment.	9,934.5	−3.108	.002	0.030	10,657.5	−1.133	.257	0.004	11,737.5	−.464	.642	0.000
22. Explained retention and stability of the prosthesis.	10,092.0	−2.819	.005	0.026	11,010.5	−.611	.541	0.002	11,359.5	−.970	.332	0.004
23. Explained expected aesthetics of the prosthesis.	10,133.5	−2.788	.005	0.024	10,974.0	−.668	.504	0.002	11,518.0	−.761	.446	0.002
24. Explained expected disadvantages of the prosthesis.	9,295.0	−3.806	<.001	0.046	11,426.0	−.027	.978	0.000	11,972.0	−.129	.897	0.000
25. Explained effects on tissues and virtual age due to prosthesis.	8,892.5	−4.293	<.001	0.060	11,216.0	−.311	.756	0.000	11,749.0	−.422	.673	0.000
26. Used appropriate language and explained technical terms.	8,783.0	−5.850	<.001	0.110	10,419.0	−1.832	.067	0.010	11,389.0	−1.182	.237	0.004
27. Encouraged and clearly answered questions.	9,366.0	−5.996	<.001	0.116	11,031.0	−.917	.359	0.002	11,724.5	−.741	.458	0.002
28. Clarified assumptions with clear facts.	8,540.0	−6.364	<.001	0.130	10,847.5	−1.084	.279	0.004	11,703.5	−.648	.517	0.002
29. Respected cultural and religious sentiments.	9,681.0	−5.437	<.001	0.094	11,236.0	−.474	.635	0.000	11,666.0	−.896	.370	0.002
30. Informed about rights in accepting/rejecting treatment plan.	8,487.5	−6.917	<.001	0.154	11,135.0	−.603	.546	0.002	11,932.5	−.260	.795	0.000
31. Satisfaction with overall treatment experience at JUH.	12,094.5	−.113	.910	0.000	11,041.5	−.682	.495	0.002	11,438.5			
	−1.040	.298	0.004									
32. Likelihood of recommending the student to others	11,205.0	−2.198	.028	0.016	11,315.0	−.310	.757	0.000	11,635.5	−.998	.318	0.004
Total professionalism scores	10,572.5	−1.998	.046	0.012	9,904.5	−1.996	.046	0.012	11,909.5	−.201	.840	0.000

JUH, Jordan University Hospital; Type of procedure, Participants received long prosthodontic procedures vs. participants received short conservative dental procedures, Students’ gender, Male vs. female dental students; Students’ study year, 4th vs. 5th year dental students, MWU, Mann Whitney *U*-test statistic; Z, Z statistic; P, Probability value; ES, Effect size using r statistic.

Table 5 demonstrates the correlations between total and individual item professionalism scores and various demographic variables including participants’ age, level of education, the distance travelled to reach the hospital, and the travel time to reach the hospital. The older participants tended to assign lower professionalism scores to the dental students (Spearman's rho test statistic = −0.112, *P* = 0.048) than the younger participants. In contrast, the time of travel to reach the hospital had no significant correlations with total or individual item professionalism scores (*P* > 0.05, Table 5). Further significant correlations between individual item scores and age, education, and distance travelled to reach the hospital are presented in Table 5.

**Table 5:** Correlations of total and individual professionalism item scores with demographic variables among the study sample (*n* = 312).

Questions	Sp rho	Age	Education	Distance	Travel time
1. First impression affected confidence in student's abilities.	r	−.081	−.050	.059	.007
	p	.155	.376	.295	.907
2. Reception at the clinic influenced confidence.	r	.005	−.028	.062	.060
	p	.933	.625	.275	.293
3. Student's attire inspired assurance.	r	.194	−.163	.119	.096
	p	.001	.004	.036	.092
4. Physical appearance/attire affected perception of future care.	r	.034	−.004	.088	.081
	p	.549	.947	.120	.154
5. Appearance and behavior exemplified professional integrity.	r	.300	−.184	.029	.057
	p	<.001	.001	.616	.316
6. Should wear a white lab coat rather than a surgical smock or scrubs.	r	−.090	.024	−.072	−.042
	p	.114	.674	.205	.458
7. Appearance/attire affected comfort level.	r	−.149	.080	−.161	−.069
	p	.008	.160	.004	.227
8. Appearance/attire affected anxiety level.	r	−.131	−.030	.040	.074
	p	.020	.595	.486	.195
9. Effective time management showed competence.	r	.165	−.182	−.062	.025
	p	.003	.001	.272	.662
10. Overall conduct conveyed competence and increased comfort.	r	.227	−.113	.044	.075
	p	<.001	.047	.443	.186
11. Student's behavior improved perception of dentists.	r	.191	-.189	−.027	.045
	p	.001	.001	.636	.426
12. Maintained infection control and orderly environment.	r	.222	−.181	.012	.033
	p	<.001	.001	.826	.564
13. Displayed professionalism comparable to faculty.	r	−.277	−.042	−.233	−.098
	p	<.001	.460	<.001	.084
14. Seemed well prepared for the procedure.	r	.267	−.249	.039	.016
	p	<.001	<.001	.496	.781
15. Demonstrated knowledge to resolve my problems.	r	.208	−.228	.084	.052
	p	<.001	<.001	.138	.362
16. Trusted student to provide the best care.	r	.182	−.177	−.019	−.051
	p	.001	.002	.738	.369
17. Chief complaint was fully heard.	r	.191	−.146	−.027	−.039
	p	.001	.010	.633	.487
18. Explained what needed to be done and why.	r	.160	−.156	.005	−.036
	p	.005	.006	.925	.526
19. Informed about diagnosis and treatment options.	r	.054	−.099	.100	.097
	p	.341	.079	.077	.088
20. Informed about treatment duration beforehand.	r	.199	−.130	.033	−.015
	p	<.001	.022	.562	.787
21. Informed about risks and benefits of treatment.	r	-.131	.026	−.079	−.048
	p	.021	.646	.162	.401
22. Explained retention and stability of the prosthesis.	r	−.166	−.035	−.060	−.034
	p	.003	.536	.292	.549
23. Explained expected aesthetics of the prosthesis.	r	−.166	−.012	−.156	−.049
	p	.003	.831	.006	.393
24. Explained expected disadvantages of the prosthesis.	r	−.221	.034	−.072	−.013
	p	<.001	.554	.207	.822
25. Explained effects on tissues and virtual age due to prosthesis.	r	−.193	.016	−.042	−.020
	p	.001	.774	.464	.725
26. Used appropriate language and explained technical terms.	r	.228	−.250	.052	.019
	p	<.001	<.001	.358	.737
27. Encouraged and clearly answered questions.	r	.209	−.188	−.030	.031
	p	<.001	.001	.602	.583
28. Clarified assumptions with clear facts.	r	.268	−.196	.045	.044
	p	<.001	.001	.425	.444
29. Respected cultural and religious sentiments.	r	.205	−.169	.006	.016
	p	<.001	.003	.922	.781
30. Informed about rights in accepting/rejecting treatment plan.	r	.211	−.173	.015	.048
	p	<.001	.002	.796	.401
31. Satisfaction with overall treatment experience at JUH.	r	.010	−.121	−.023	−.027
	p	.867	.032	.685	.630
32. Likelihood of recommending the student to others	r	.119	−.134	.029	.032
	p	.035	.018	.604	.572
Total professionalism scores	R	−.112	−.065	−.070	−.015
	p	.048	.253	.217	.789

Sp rho, Spearman's rho correlation test; R, Spearman's Rho correlation coefficient; P, Probability value using Spearman's Rho correlation test; JUH; Jordan University Hospital.

Table 6 demonstrates the hierarchical multiple linear regression analysis of total professionalism scores using the study year of the student, the gender of the student, and the type of received procedure; while controlling for age, education, travel time, and travel distance. Higher levels of participants' education were associated with lower levels of professionalism scores (*B* = −1.106, 95%CI of *B* = −2.187–−0.025, *P* = 0.045, R^2^ = 0.034, Table 6). In addition, a stepwise multiple linear regression analysis of total professionalism scores starting with factors including the study year of the student, the gender of the student, the type of procedure received, age, education, travel time, and travel distance revealed that being a female dental student was associated with higher professionalism scores than being male (*B* = 3.292, 95%CI of *B* = 0.171–6.413, *P* = 0.039, R^2^ = 0.014; Table 6).

**Table 6:** Multiple linear regression analysis to predict total professionalism scores among the study sample (*n* = 312).

Dependent variable	Predictors	Unstand Co		Stand Co	*t*	*P*	95% CI for B		Collinearity test	
		B	SE	Beta			Lower Bound	Upper Bound	Tol	VIF
Regression for total professionalism scores (TPS) using hierarchical regression analysis^a^										
TPS R^2^ = .034	(Constant)	145.193	7.355	—	19.740	<.001	130.719	159.666	—	—
DW = 1.964	Age	−.469	1.313	−.029	−.358	.721	−3.052	2.114	.485	2.063
	education	−1.106	.549	−.119	−2.013	.045	−2.187	−.025	.915	1.093
	Travel distance	−1.926	1.348	−.106	−1.429	.154	−4.577	.726	.573	1.746
	Travel time	.223	1.088	.015	.205	.838	−1.918	2.365	.580	1.725
	Type of dental procedure	.193	2.229	.007	.087	.931	−4.193	4.579	.476	2.102
	Year of dental student	−.579	1.535	−.021	−.377	.706	−3.600	2.442	.980	1.020
	Gender of dental students	3.012	1.595	.107	1.888	.060	−.127	6.150	.987	1.013
Regression for total professionalism scores using stepwise regression analysis										
TPS R^2^ = .014	(Constant)	135.318	2.685	—	50.396	<.001	130.035	140.602	—	—
DW = 1.969	Gender of dental students	3.292	1.586	.117	2.075	.039	.171	6.413	1.000	1.000

R^2^, Coefficient of determination; DW, Durbin-Watson test statistic; Unstand Co, Unstandardized coefficient; Stand Co, Standardized coefficient; B, Beta statistics; SE, Standard Error; t, t statistics; P, Two tailed probability value; CI, Confidence intervals; Tol, Tolerance; VIF, Variance inflation factor.

a Final model is presented with age, education, travel time, and travel distance were included in the first block of the regression model, while the type of dental procedure, gender of student, and student's year of study were included in the second block.

## 4 Discussion

This study evaluated dental students' professionalism from the patients' perspective in fixed and removable prosthodontic clinics at the School of Dentistry, The University of Jordan. Overall, patients reported high levels of professionalism, with most items receiving high scores and low variability, indicating consistently positive perceptions. The findings of this study showed that patients highly rated the students' professionalism. This finding is consistent with previous studies reporting generally positive patient perceptions of dental students' professionalism. This aligns with recent evidence emphasizing the importance of communication and interpersonal skills in shaping patient satisfaction and trust (9, 10). However, statistically significant differences were observed based on treatment type and student gender. These findings highlight the importance of reinforcing professional behaviour across clinical training in dental education.

Most of the responses to individual items had a large median (5) with a small interquartile range indicating that the participants tended to assign high scores for student professionalism in this investigation. Professionalism scores were higher for short-term procedures compared with long-term treatments. Although, this difference was associated with small effect sizes and weak correlations. Fewer visits may lead to more focused and professional interactions, professionalism has multiple dimensions, such as communication, empathy, and clinical competence (20), which could explain the higher rates of professionalism. On the other hand, longer treatments may introduce variability in the patient experience. Professionalism may fluctuate due to the complexity of the treatment and the differing interactions with students over time (21). In addition, patient expectations, previous dental experiences, and other individual factors could also impact how professionalism is assessed (22). Therefore, the higher professionalism ratings for short-term procedures likely reflect a combination of treatment simplicity, focused interactions, and patient satisfaction rather than just the number of visits. Effective communication plays an important role in patients' evaluation of professionalism, especially, in shorter and more focused clinical encounters (9). Nevertheless, this finding should be interpreted with caution.

Professionalism scores were higher for female students compared with male students. However, the regression analysis demonstrated very limited explanatory power (R² < 0.05), indicating that gender is a weak predictor of perceived professionalism. This finding may reflect differences in patient perceptions of communication style or interpersonal behaviour, as suggested in previous literature. However, it should be interpreted cautiously, as such differences may also be influenced by sociocultural expectations or implicit bias rather than actual differences in professional competence. Female students tend to be responsible and to show empathetic behaviour (14). Patients' preferences for dentist gender may also be influenced by their personality characteristics, previous dental experiences, cultural background, and personal comfort levels (23). Traditional perceptions often suggest that male dentists are categorized as more competent, whereas female dentists are regarded as more empathetic (24).This observation is consistent with previous research suggesting that communication style and emotional intelligence may influence patient perceptions of professionalism (10). However, such findings should be interpreted cautiously, as they may also reflect sociocultural expectations rather than objective differences in professional competence.

Differences between 4th- and 5th-year students were inconsistent. While 4th-year students scored higher on selected interpersonal items, 5th-year students showed comparable or slightly higher ratings across most other domains. These findings suggest that academic year alone is not a strong determinant of perceived professionalism and may reflect differences in clinical exposure, supervision, or patient expectations. Although 5th-year students generally have more clinical experience, the 4th-year students may have shown higher professionalism in specific areas owing to their recent transition into clinical training and high motivation to make a strong first impression on patients and faculty. Furthermore, the transition from preclinical to clinical education allows the students to adhere more to the established standards and protocols of professional conduct, such as dress code, demeanor, and patient communication protocols (25). Another explanation could be that students are at a closer faculty observation and monitoring of their interactions with patients during their early clinical training (26). Thus, the students might be more encouraged to focus on aspects such as attire, body language, active listening, and empathy, which significantly showed their professionalism (27).

At the item level, differences between short- and long-term procedures suggest that patients may evaluate different dimensions of professionalism depending on the clinical context. Long-term treatments may allow better assessment of continuity of care and information sharing, whereas short-term procedures may emphasise immediate communication and patient comfort. The patient's initial impression is believed to be affected by three aspects: the way the patient is greeted in the first appointment, the way the clinicians maintain themselves, and the reception area environment (19). In a recent study 90% of the patients believed that the dentist outlook and their clothing significantly affected their evaluation of the dentists' competency and confidence. Similarly, most patients believe that the dental students' treatment positively influenced their confidence in the treatment provided (19).

Sociodemographic variables showed weak but statistically significant associations with professionalism scores. Older and more educated patients tended to assign slightly lower ratings, possibly reflecting differing expectations. Travel-related factors may also influence patient perceptions indirectly through their impact on access to care and overall treatment experience. However, these associations were modest and should be interpreted cautiously. Socio-demographic variables could influence patients' perception of health care (28). For instance, older patients reported higher health care level than the younger patients did (29); 60% of the participants were above 40 years of age, which can affect the patients' response to different genders (30). Patients' educational level could influence their ability to evaluate the students' professionalism. The participants with lower educational levels reported higher health care levels than did those with higher educational levels. This could be attributed to the higher expectations of highly educated patients (29). Travel time and distance may indirectly influence patient perceptions of dental student professionalism by affecting healthcare interactions, patient stress levels, and students' professional performance. Recognizing these factors highlights the complex relationship between external conditions and the assessment of professional competence (31). When dental care is accessible and stress-free, patients are more likely to return and build long-term trust with their dentists (32).

While this study provides valuable insights, it has several limitations. Since it was conducted over a short period, it lacks the depth that a long-term study could offer. The wide age range of participants also makes it challenging to link patient evaluations to specific age-related factors. Additionally, being limited to a single university reduces the ability to generalize the findings, and using a Likert scale may have restricted the richness of patient responses (33). Therefore, future research should follow a more comprehensive approach that could involve multiple institutions nationally or internationally. Biases could also arise from patients' varied appointment experiences, voluntary participation, and personal perceptions of professionalism, potentially influencing the results. To build on this study, future research should involve multiple institutions and use a mix of surveys and interviews to gain deeper insights into dental students' professionalism.

A high response rate of 89.1% was achieved, with 312 patients voluntarily completing the survey during the 2022–2023 academic year. This approach allowed for efficient data collection and included diverse samples across different age groups and educational backgrounds. However, as a non-probability sampling method, it introduces potential selection bias, limiting the generalizability of the findings beyond this specific clinical setting. While the study provides valuable insights into patient perceptions of dental student professionalism, the results primarily reflect the experiences of those who received treatment at this hospital.

Patient-based assessments of dental student professionalism are inherently subjective and may be influenced by personal experiences, expectations, and cultural factors. Additionally, response bias and social desirability bias can impact the reliability of evaluations, where patients may feel inclined to rate the students more favourably or unfavourably to align with societal expectations of professionalism, rather than offering an objective assessment. To enhance validity of the findings, the research methodologies incorporated strategies such as anonymous surveys, multi-source feedback, and structured assessment frameworks. A balanced approach of patient perspectives and objective evaluation methods was implemented to ensure a comprehensive understanding of professional competence in dental education.

In general, G*Power is applied to studies using probability sampling. However, when it is required to ensure adequate statistical power, it is also broadly applied in non-probability sampling research (34, 35). The regression model explained less than 5% of the variance in professionalism scores, highlighting the limited explanatory power of the included variables. This suggests that professionalism is influenced by additional factors not captured in this study, such as individual student characteristics (e.g., communication skills, personality traits), clinical environment factors (e.g., supervision and workload), and patient-related variables (e.g., expectations and prior experiences). Further research is recommended to explore additional underlying factors that might contribute to the professionalism scores in this regard. Further, most responses had a median score of 5 with a narrow interquartile range suggests a potential ceiling effect which could be due to social desirability bias for instance, respondents sometimes choose the highest option as a result of their desire to provide positive feedback. This is common in patient surveys and satisfaction studies.

Despite these limitations, this study provides valuable insights into patient-based evaluations of dental student professionalism in a clinical educational setting. A key strength is the inclusion of real-time patient feedback across different treatment types. Future research should adopt multi-institutional and mixed-method approaches to better capture the complexity of professionalism. Incorporating perspectives from patients, faculty, and peers may provide a more comprehensive evaluation framework. The findings of this study highlight the importance of integrating structured professionalism and communication training into dental curricula. Further, patient feedback integration into assessment strategies could support the development of professionalism in clinical training.

## 5 Conclusions

The dental students received high scores for professionalism in this investigation reflecting a high level of professionalism in the clinical years. Patients' perceptions of their professionalism are influenced by sociodemographic factors such as patients' age and educational level as well as by sociodemographic factors of dental students such as gender and year of study. Furthermore, it can be influenced by the length of treatment (short- or long-term dental procedures).

## Data Availability

The original contributions presented in the study are included in the article/supplementary material, further inquiries can be directed to the corresponding author/s.
