# Supplementary material for: Patient perceptions of dental students' professionalism in undergraduate prosthodontic clinics: a cross-sectional study
Source: Front Oral Health. 2026 Apr 24;7:1802263. doi: 10.3389/froh.2026.1802263 (PMC13154600; doi:10.3389/froh.2026.1802263)
Supplement: Supplementary file 1 [file Supplementaryfile1.docx]

# Appendices

**Appendix 1:** Content validity index for items and scale for the survey used in this study.

| Item | Tested factor | Experts’ feedback* | | | Number of agreements between experts^$^ | I-CVI |
| --- | --- | --- | --- | --- | --- | --- |
|  |  | Expert  1 | Expert  2 | Expert  3 |  |  |
| 1. First impression affected confidence in student abilities | Relevance | 4 | 4 | 4 | 3 | 1 |
|  | Clarity | 4 | 4 | 4 | 3 | 1 |
| 2. Reception in clinic affected confidence level | Relevance | 4 | 4 | 4 | 3 | 1 |
|  | Clarity | 4 | 4 | 4 | 3 | 1 |
| 3. Students’ attire assured their abilities | Relevance | 4 | 4 | 4 | 3 | 1 |
|  | Clarity | 4 | 4 | 4 | 3 | 1 |
| 4. Student’s appearance affected feelings about future care | Relevance | 4 | 4 | 4 | 3 | 1 |
|  | Clarity | 4 | 4 | 4 | 3 | 1 |
| 5. Student’s appearance and behaviors exemplified professional integrity | Relevance | 4 | 4 | 4 | 3 | 1 |
|  | Clarity | 4 | 4 | 4 | 3 | 1 |
| 6. Should wear a white lab coat not surgical scrubs | Relevance | 4 | 4 | 4 | 3 | 1 |
|  | Clarity | 4 | 4 | 4 | 3 | 1 |
| 7. Student’s appearance /attire greatly affected my comfort | Relevance | 4 | 4 | 4 | 3 | 1 |
|  | Clarity | 4 | 4 | 4 | 3 | 1 |
| 8. Student’s appearance /attire greatly affected my anxiety | Relevance | 4 | 4 | 4 | 3 | 1 |
|  | Clarity | 4 | 4 | 4 | 3 | 1 |
| 9. Effective time management showed student competence | Relevance | 4 | 4 | 4 | 3 | 1 |
|  | Clarity | 4 | 4 | 4 | 3 | 1 |
| 10. Student conduct conveyed competence and increased comfort | Relevance | 4 | 4 | 4 | 3 | 1 |
|  | Clarity | 4 | 4 | 4 | 3 | 1 |
| 11. Student behavior improved perception of dentists in general | Relevance | 4 | 4 | 4 | 3 | 1 |
|  | Clarity | 4 | 4 | 4 | 3 | 1 |
| 12. Student kept adequate infection control and orderly work environment | Relevance | 4 | 4 | 4 | 3 | 1 |
|  | Clarity | 4 | 4 | 4 | 3 | 1 |
| 13. Student professionalism equals faculty /professors | Relevance | 4 | 4 | 4 | 3 | 1 |
|  | Clarity | 4 | 4 | 4 | 3 | 1 |
| 14. Student well prepared for today’s procedure | Relevance | 4 | 4 | 4 | 3 | 1 |
|  | Clarity | 4 | 4 | 4 | 3 | 1 |
| 15. Student has knowledge to resolve my problems | Relevance | 4 | 4 | 4 | 3 | 1 |
|  | Clarity | 4 | 4 | 4 | 3 | 1 |
| 16. Student took care of me in the best way possible | Relevance | 4 | 4 | 4 | 3 | 1 |
|  | Clarity | 4 | 4 | 4 | 3 | 1 |
| 17. My chief complaint was heard fully and in detail | Relevance | 4 | 4 | 4 | 3 | 1 |
|  | Clarity | 4 | 4 | 4 | 3 | 1 |
| 18. Student explained what needed to be done and why | Relevance | 4 | 4 | 4 | 3 | 1 |
|  | Clarity | 4 | 4 | 4 | 3 | 1 |
| 19. Informed about the diagnosis and treatments | Relevance | 4 | 4 | 4 | 3 | 1 |
|  | Clarity | 4 | 4 | 4 | 3 | 1 |
| 20. Informed about treatment duration before treatment | Relevance | 4 | 4 | 4 | 3 | 1 |
|  | Clarity | 4 | 4 | 4 | 3 | 1 |
| 21. Informed about risks and benefits of treatment | Relevance | 4 | 4 | 4 | 3 | 1 |
|  | Clarity | 4 | 4 | 4 | 3 | 1 |
| 22. Informed about prosthesis expected retention/ stability | Relevance | 4 | 4 | 4 | 3 | 1 |
|  | Clarity | 4 | 4 | 4 | 3 | 1 |
| 23. Informed about prosthesis expected aesthetics | Relevance | 4 | 4 | 4 | 3 | 1 |
|  | Clarity | 4 | 4 | 4 | 3 | 1 |
| 24. Informed about prosthesis expected disadvantages | Relevance | 4 | 4 | 4 | 3 | 1 |
|  | Clarity | 4 | 4 | 4 | 3 | 1 |
| 25. Informed about prosthesis tissue effects and virtual age | Relevance | 4 | 4 | 4 | 3 | 1 |
|  | Clarity | 4 | 4 | 4 | 3 | 1 |
| 26. Student used appropriate language and explained technical terms effectively | Relevance | 4 | 4 | 4 | 3 | 1 |
|  | Clarity | 4 | 4 | 4 | 3 | 1 |
| 27. Questions encouraged and clearly answered | Relevance | 4 | 4 | 4 | 3 | 1 |
|  | Clarity | 4 | 4 | 4 | 3 | 1 |
| 28. My assumptions were clarified by clear facts | Relevance | 4 | 4 | 4 | 3 | 1 |
|  | Clarity | 4 | 4 | 4 | 3 | 1 |
| 29. My culture and religious sentiments were respected | Relevance | 4 | 4 | 4 | 3 | 1 |
|  | Clarity | 4 | 4 | 4 | 3 | 1 |
| 30. Informed about my rights in accepting or rejecting the treatment plan | Relevance | 4 | 4 | 4 | 3 | 1 |
|  | Clarity | 4 | 4 | 4 | 3 | 1 |
| 31. Overall satisfaction with treatment experience you get from dental students in JUH | Relevance | 4 | 4 | 4 | 3 | 1 |
|  | Clarity | 4 | 4 | 4 | 3 | 1 |
| 32. How likely you would recommend him/her to others | Relevance | 3 | 3 | 4 | 3 | 1 |
|  | Clarity | 3 | 4 | 3 | 3 | 1 |
| All items: S-CVI | Relevance | 1 | | | | |
|  | Clarity | 1 | | | | |

*Expert feedback for relevance was not relevant (score 1), somewhat relevant (score 2), quite relevant (score 3), and highly relevant (score 4); and for Clarity was not clear (score 1), need significant change/revision to be clear (score 2), need minor change/revision to be clear (score 3), and very clear (score 4). ^$^Number of scores ≥3 of the experts’ feedback, I-CVI= Item content validity index, S-CVI= Scale content validity index.

**Appendix 2:** Distribution of frequencies and percentages of participants’ responses to individual items regarding dental students’ professionalism among the study population (n= 312).

| *Question Item number | | Total sample (n= 312) | | | | | Prosthodontics (n= 159) | | | | | Conservative (n= 153) | | | | |
| --- | --- | --- | --- | --- | --- | --- | --- | --- | --- | --- | --- | --- | --- | --- | --- | --- |
|  |  | SD | D | Neu | A | SA | SD | D | Neu | A | SA | SD | D | Neu | A | SA |
| 1 | N | 4 | 33 | 13 | 95 | 167 | 3 | 25 | 6 | 42 | 83 | 1 | 8 | 7 | 53 | 84 |
|  | % | 1.3 | 10.6 | 4.2 | 30.4 | 53.5 | 1.9 | 15.7 | 3.8 | 26.4 | 52.2 | 0.7 | 5.2 | 4.6 | 34.6 | 54.9 |
| 2 | N | 3 | 18 | 5 | 80 | 206 | 2 | 13 | 1 | 35 | 108 | 1 | 5 | 4 | 45 | 98 |
|  | % | 1.0 | 5.8 | 1.6 | 25.6 | 66.0 | 1.3 | 8.2 | 0.6 | 22.0 | 67.9 | 0.7 | 3.3 | 2.6 | 29.4 | 64.1 |
| 3 | N | 3 | 3 | 17 | 71 | 218 | 0 | 1 | 4 | 26 | 128 | 3 | 2 | 13 | 45 | 90 |
|  | % | 1.0 | 1.0 | 5.4 | 22.8 | 69.9 | 0 | 0.6 | 2.5 | 16.4 | 80.5 | 2.0 | 1.3 | 8.5 | 29.4 | 58.8 |
| 4 | N | 5 | 23 | 39 | 63 | 182 | 1 | 15 | 20 | 24 | 99 | 4 | 8 | 19 | 39 | 83 |
|  | % | 1.6 | 7.4 | 12.5 | 20.2 | 58.3 | .6 | 9.4 | 12.6 | 15.1 | 62.3 | 2.6 | 5.2 | 12.4 | 25.5 | 54.2 |
| 5 | N | 2 | 4 | 8 | 58 | 240 | 0 | 0 | 1 | 13 | 145 | 2 | 4 | 7 | 45 | 95 |
|  | % | .6 | 1.3 | 2.6 | 18.6 | 76.9 | 0 | 0 | .6 | 8.2 | 91.2 | 1.3 | 2.6 | 4.6 | 29.4 | 62.1 |
| 6 | N | 30 | 28 | 124 | 53 | 77 | 20 | 13 | 72 | 13 | 41 | 10 | 15 | 52 | 40 | 36 |
|  | % | 9.6 | 9.0 | 39.7 | 17.0 | 24.7 | 12.6 | 8.2 | 45.3 | 8.2 | 25.8 | 6.6 | 9.8 | 34.0 | 26.1 | 23.5 |
| 7 | N | 3 | 9 | 98 | 61 | 141 | 0 | 5 | 77 | 18 | 59 | 3 | 4 | 21 | 43 | 82 |
|  | % | 1.0 | 2.9 | 31.4 | 19.6 | 45.2 | 0 | 3.1 | 48.4 | 11.3 | 37.1 | 2.0 | 2.6 | 13.7 | 28.1 | 53.6 |
| 8 | N | 46 | 75 | 111 | 31 | 49 | 20 | 39 | 82 | 7 | 11 | 26 | 36 | 29 | 24 | 38 |
|  | % | 14.8 | 24.0 | 35.6 | 9.9 | 15.7 | 12.6 | 24.5 | 51.6 | 4.4 | 6.9 | 17 | 23.5 | 19.0 | 15.7 | 24.8 |
| 9 | N | 2 | 12 | 19 | 68 | 211 | 0 | 7 | 7 | 18 | 127 | 2 | 5 | 12 | 50 | 84 |
|  | % | .6 | 3.8 | 6.1 | 21.8 | 67.6 | 0 | 4.4 | 4.4 | 11.3 | 79.9 | 1.3 | 3.3 | 7.8 | 32.7 | 54.9 |
| 10 | N | 0 | 6 | 16 | 53 | 237 | 0 | 2 | 2 | 12 | 143 | 0 | 4 | 14 | 41 | 94 |
|  | % | 0 | 1.9 | 5.1 | 17.0 | 76.0 | 0 | 1.3 | 1.3 | 7.5 | 89.9 | 0 | 2.6 | 9.2 | 26.8 | 61.4 |
| 11 | N | 0 | 5 | 29 | 57 | 221 | 0 | 1 | 16 | 9 | 133 | 0 | 4 | 13 | 48 | 88 |
|  | % | 0 | 1.6 | 9.3 | 18.3 | 70.8 | 0 | .6 | 10.1 | 5.7 | 83.6 | 0 | 2.6 | 8.5 | 31.4 | 57.5 |
| 12 | N | 0 | 2 | 9 | 47 | 254 | 0 | 0 | 2 | 7 | 150 | 0 | 2 | 7 | 40 | 104 |
|  | % | 0 | .6 | 2.9 | 15.1 | 81.4 | 0 | 0 | 1.3 | 4.4 | 94.3 | 0 | 1.3 | 4.6 | 26.1 | 68.0 |
| 13 | N | 48 | 25 | 35 | 79 | 125 | 48 | 17 | 23 | 20 | 51 | 0 | 8 | 12 | 59 | 74 |
|  | % | 15.4 | 8.0 | 11.2 | 25.3 | 40.1 | 30.1 | 10.7 | 14.5 | 12.6 | 32.1 | 0 | 5.2 | 7.8 | 38.6 | 48.4 |
| 14 | N | 1 | 5 | 8 | 59 | 239 | 0 | 2 | 1 | 7 | 149 | 1 | 3 | 7 | 52 | 90 |
|  | % | .3 | 1.6 | 2.6 | 18.9 | 76.6 | 0 | 1.3 | .6 | 4.4 | 93.7 | .7 | 2.0 | 4.6 | 34.0 | 58.8 |
| 15 | N | 0 | 8 | 10 | 78 | 216 | 0 | 3 | 3 | 22 | 131 | 0 | 5 | 7 | 56 | 85 |
|  | % | 0 | 2.6 | 3.2 | 25.0 | 69.2 | 0 | 1.9 | 1.9 | 13.8 | 82.4 | 0 | 3.3 | 4.6 | 36.6 | 55.6 |
| 16 | N | 1 | 5 | 9 | 52 | 245 | 1 | 2 | 5 | 10 | 141 | 0 | 3 | 4 | 42 | 104 |
|  | % | .3 | 1.6 | 2.9 | 16.7 | 78.5 | .6 | 1.3 | 3.1 | 6.3 | 88.7 | 0 | 2.0 | 2.6 | 27.5 | 68.0 |
| 17 | N | 0 | 2 | 3 | 36 | 271 | 0 | 1 | 0 | 3 | 155 | 0 | 1 | 3 | 33 | 116 |
|  | % | 0 | .6 | 1.0 | 11.5 | 86.9 | 0 | .6 | 0 | 1.9 | 97.5 | 0 | .7 | 2.0 | 21.6 | 75.8 |
| 18 | N | 2 | 0 | 4 | 54 | 252 | 2 | 0 | 3 | 8 | 146 | 0 | 0 | 1 | 46 | 106 |
|  | % | .6 | 0 | 1.3 | 17.3 | 80.8 | 1.3 | 0 | 1.9 | 5.0 | 91.8 | 0 | 0 | .7 | 30.1 | 69.3 |
| 19 | N | 5 | 9 | 5 | 57 | 236 | 5 | 7 | 1 | 18 | 128 | 0 | 2 | 4 | 39 | 108 |
|  | % | 1.6 | 2.9 | 1.6 | 18.3 | 75.6 | 3.1 | 4.4 | .6 | 11.3 | 80.5 | 0 | 1.3 | 2.6 | 25.5 | 70.6 |
| 20 | N | 7 | 12 | 20 | 39 | 234 | 7 | 4 | 3 | 6 | 139 | 0 | 8 | 17 | 33 | 95 |
|  | % | 2.2 | 3.8 | 6.4 | 12.5 | 75.0 | 4.4 | 2.5 | 1.9 | 3.8 | 87.4 | 0 | 5.2 | 11.1 | 21.6 | 62.1 |
| 21 | N | 37 | 13 | 18 | 69 | 175 | 34 | 9 | 5 | 30 | 81 | 3 | 4 | 13 | 39 | 94 |
|  | % | 11.9 | 4.2 | 5.8 | 22.1 | 56.1 | 21.4 | 5.7 | 3.1 | 18.9 | 50.9 | 2.0 | 2.6 | 8.5 | 25.5 | 61.4 |
| 22 | N | 32 | 19 | 17 | 88 | 156 | 27 | 15 | 4 | 41 | 72 | 5 | 4 | 13 | 47 | 84 |
|  | % | 10.3 | 6.1 | 5.4 | 28.2 | 50.0 | 17.0 | 9.4 | 2.5 | 25.8 | 45.3 | 3.3 | 2.6 | 8.5 | 30.7 | 54.9 |
| 23 | N | 36 | 15 | 16 | 81 | 164 | 32 | 7 | 4 | 41 | 75 | 4 | 8 | 12 | 40 | 89 |
|  | % | 11.5 | 4.8 | 5.1 | 26.0 | 52.6 | 20.1 | 4.4 | 2.5 | 25.8 | 47.2 | 2.6 | 5.2 | 7.8 | 26.1 | 58.2 |
| 24 | N | 37 | 40 | 19 | 79 | 137 | 32 | 26 | 4 | 38 | 59 | 5 | 14 | 15 | 41 | 78 |
|  | % | 11.9 | 12.8 | 6.1 | 25.3 | 43.9 | 20.1 | 16.4 | 2.5 | 23.9 | 37.1 | 3.3 | 9.2 | 9.8 | 26.8 | 51.0 |
| 25 | N | 38 | 39 | 28 | 84 | 123 | 34 | 26 | 6 | 43 | 50 | 4 | 13 | 22 | 41 | 73 |
|  | % | 12.2 | 12.5 | 9.0 | 26.9 | 39.4 | 21.4 | 16.4 | 3.8 | 27.0 | 31.4 | 2.6 | 8.5 | 14.4 | 26.8 | 47.7 |
| 26 | N | 1 | 3 | 6 | 60 | 242 | 0 | 1 | 1 | 12 | 145 | 1 | 2 | 5 | 48 | 97 |
|  | % | .3 | 1.0 | 1.9 | 19.2 | 77.6 | 0 | .6 | .6 | 7.5 | 91.2 | .7 | 1.3 | 3.3 | 31.4 | 63.4 |
| 27 | N | 0 | 1 | 3 | 37 | 271 | 0 | 0 | 0 | 3 | 156 | 0 | 1 | 3 | 34 | 115 |
|  | % | 0 | .3 | 1.0 | 11.9 | 86.9 | 0 | 0 | 0 | 1.9 | 98.1 | 0 | .7 | 2.0 | 22.2 | 75.2 |
| 28 | N | 1 | 2 | 11 | 53 | 245 | 0 | 0 | 2 | 9 | 148 | 1 | 2 | 9 | 44 | 97 |
|  | % | .3 | .6 | 3.5 | 17.0 | 78.5 | 0 | 0 | 1.3 | 5.7 | 93.1 | .7 | 1.3 | 5.9 | 28.8 | 63.4 |
| 29 | N | 0 | 0 | 3 | 36 | 273 | 0 | 0 | 0 | 4 | 155 | 0 | 0 | 3 | 32 | 118 |
|  | % | 0 | 0 | 1.0 | 11.5 | 87.5 | 0 | 0 | 0 | 2.5 | 97.5 | 0 | 0 | 2.0 | 20.9 | 77.1 |
| 30 | N | 1 | 6 | 7 | 42 | 256 | 0 | 1 | 0 | 4 | 154 | 1 | 5 | 7 | 38 | 102 |
|  | % | .3 | 1.9 | 2.2 | 13.5 | 82.1 | 0 | .6 | 0 | 2.5 | 96.9 | .7 | 3.3 | 4.6 | 24.8 | 66.7 |
| 31 | N | 0 | 3 | 2 | 78 | 229 | 0 | 1 | 1 | 40 | 117 | 0 | 2 | 1 | 38 | 112 |
|  | % | 0 | 1.0 | .6 | 25.0 | 73.4 | 0 | .6 | .6 | 25.2 | 73.6 | 0 | 1.3 | .7 | 24.8 | 73.2 |
| 32 | N | 0 | 5 | 8 | 22 | 277 | 0 | 0 | 2 | 10 | 147 | 0 | 5 | 6 | 12 | 130 |
|  | % | 0 | 1.6 | 2.6 | 7.1 | 88.8 | 0 | 0 | 1.3 | 6.3 | 92.5 | 0 | 3.3 | 3.9 | 7.8 | 85.0 |

*The label for each item is presented in Table 3. Prosthodontic= Participants received long prosthodontic procedures, Conservative= Participants received short conservative dental procedures. SD: strongly disagree, D: disagree, Neu: neutral, A: agree, SA: strongly agree

**Appendix 2:** Assessment of Eigenvalues of item loading on the total professionalism score using confirmatory factor analysis for items of the survey used in this study.

| Item | Eigenvalues* |
| --- | --- |
| 1. First impression affected confidence in student's abilities. | .682 |
| 2. Reception at the clinic influenced confidence. | .640 |
| 3. Student's attire inspired assurance. | .675 |
| 4. Physical appearance/attire affected perception of future care. | .625 |
| 5. Appearance and behavior exemplified professional integrity. | .576 |
| 6. Should wear a white lab coat rather than a surgical smock or scrubs. | .427 |
| 7. Appearance/attire affected comfort level. | .632 |
| 8. Appearance/attire affected anxiety level. | .486 |
| 9. Effective time management showed competence. | .627 |
| 10. Overall conduct conveyed competence and increased comfort. | .587 |
| 11. Student’s behavior improved perception of dentists. | .653 |
| 12. Maintained infection control and orderly environment. | .728 |
| 13. Displayed professionalism comparable to faculty. | .576 |
| 14. Seemed well prepared for the procedure. | .670 |
| 15. Demonstrated knowledge to resolve my problems. | .707 |
| 16. Trusted student to provide the best care. | .651 |
| 17. Chief complaint was fully heard. | .499 |
| 18. Explained what needed to be done and why. | .750 |
| 19. Informed about diagnosis and treatment options. | .478 |
| 20. Informed about treatment duration beforehand. | .523 |
| 21. Informed about risks and benefits of treatment. | .754 |
| 22. Explained retention and stability of the prosthesis. | .734 |
| 23. Explained expected aesthetics of the prosthesis. | .837 |
| 24. Explained expected disadvantages of the prosthesis. | .832 |
| 25. Explained effects on tissues and virtual age due to prosthesis. | .805 |
| 26. Used appropriate language and explained technical terms. | .608 |
| 27. Encouraged and clearly answered questions. | .687 |
| 28. Clarified assumptions with clear facts. | .628 |
| 29. Respected cultural and religious sentiments. | .665 |
| 30. Informed about rights in accepting/rejecting treatment plan. | .650 |
| 31. Satisfaction with overall treatment experience at JUH. | .736 |
| 32. Likelihood of recommending the student to others | .674 |

*Extraction method is principal component analysis with initial eigenvalue of 1.

**Appendix 3:** Distribution of frequencies and percentages of participants’ responses to individual items regarding dental students’ professionalism among the study population (n= 312).

| *Question Item number | | Total sample (n= 312) | | | | | Prosthodontics (n= 159) | | | | | Conservative (n= 153) | | | | |
| --- | --- | --- | --- | --- | --- | --- | --- | --- | --- | --- | --- | --- | --- | --- | --- | --- |
|  |  | SD | D | Neu | A | SA | SD | D | Neu | A | SA | SD | D | Neu | A | SA |
| 1 | N | 4 | 33 | 13 | 95 | 167 | 3 | 25 | 6 | 42 | 83 | 1 | 8 | 7 | 53 | 84 |
|  | % | 1.3 | 10.6 | 4.2 | 30.4 | 53.5 | 1.9 | 15.7 | 3.8 | 26.4 | 52.2 | 0.7 | 5.2 | 4.6 | 34.6 | 54.9 |
| 2 | N | 3 | 18 | 5 | 80 | 206 | 2 | 13 | 1 | 35 | 108 | 1 | 5 | 4 | 45 | 98 |
|  | % | 1.0 | 5.8 | 1.6 | 25.6 | 66.0 | 1.3 | 8.2 | 0.6 | 22.0 | 67.9 | 0.7 | 3.3 | 2.6 | 29.4 | 64.1 |
| 3 | N | 3 | 3 | 17 | 71 | 218 | 0 | 1 | 4 | 26 | 128 | 3 | 2 | 13 | 45 | 90 |
|  | % | 1.0 | 1.0 | 5.4 | 22.8 | 69.9 | 0 | 0.6 | 2.5 | 16.4 | 80.5 | 2.0 | 1.3 | 8.5 | 29.4 | 58.8 |
| 4 | N | 5 | 23 | 39 | 63 | 182 | 1 | 15 | 20 | 24 | 99 | 4 | 8 | 19 | 39 | 83 |
|  | % | 1.6 | 7.4 | 12.5 | 20.2 | 58.3 | .6 | 9.4 | 12.6 | 15.1 | 62.3 | 2.6 | 5.2 | 12.4 | 25.5 | 54.2 |
| 5 | N | 2 | 4 | 8 | 58 | 240 | 0 | 0 | 1 | 13 | 145 | 2 | 4 | 7 | 45 | 95 |
|  | % | .6 | 1.3 | 2.6 | 18.6 | 76.9 | 0 | 0 | .6 | 8.2 | 91.2 | 1.3 | 2.6 | 4.6 | 29.4 | 62.1 |
| 6 | N | 30 | 28 | 124 | 53 | 77 | 20 | 13 | 72 | 13 | 41 | 10 | 15 | 52 | 40 | 36 |
|  | % | 9.6 | 9.0 | 39.7 | 17.0 | 24.7 | 12.6 | 8.2 | 45.3 | 8.2 | 25.8 | 6.6 | 9.8 | 34.0 | 26.1 | 23.5 |
| 7 | N | 3 | 9 | 98 | 61 | 141 | 0 | 5 | 77 | 18 | 59 | 3 | 4 | 21 | 43 | 82 |
|  | % | 1.0 | 2.9 | 31.4 | 19.6 | 45.2 | 0 | 3.1 | 48.4 | 11.3 | 37.1 | 2.0 | 2.6 | 13.7 | 28.1 | 53.6 |
| 8 | N | 46 | 75 | 111 | 31 | 49 | 20 | 39 | 82 | 7 | 11 | 26 | 36 | 29 | 24 | 38 |
|  | % | 14.8 | 24.0 | 35.6 | 9.9 | 15.7 | 12.6 | 24.5 | 51.6 | 4.4 | 6.9 | 17 | 23.5 | 19.0 | 15.7 | 24.8 |
| 9 | N | 2 | 12 | 19 | 68 | 211 | 0 | 7 | 7 | 18 | 127 | 2 | 5 | 12 | 50 | 84 |
|  | % | .6 | 3.8 | 6.1 | 21.8 | 67.6 | 0 | 4.4 | 4.4 | 11.3 | 79.9 | 1.3 | 3.3 | 7.8 | 32.7 | 54.9 |
| 10 | N | 0 | 6 | 16 | 53 | 237 | 0 | 2 | 2 | 12 | 143 | 0 | 4 | 14 | 41 | 94 |
|  | % | 0 | 1.9 | 5.1 | 17.0 | 76.0 | 0 | 1.3 | 1.3 | 7.5 | 89.9 | 0 | 2.6 | 9.2 | 26.8 | 61.4 |
| 11 | N | 0 | 5 | 29 | 57 | 221 | 0 | 1 | 16 | 9 | 133 | 0 | 4 | 13 | 48 | 88 |
|  | % | 0 | 1.6 | 9.3 | 18.3 | 70.8 | 0 | .6 | 10.1 | 5.7 | 83.6 | 0 | 2.6 | 8.5 | 31.4 | 57.5 |
| 12 | N | 0 | 2 | 9 | 47 | 254 | 0 | 0 | 2 | 7 | 150 | 0 | 2 | 7 | 40 | 104 |
|  | % | 0 | .6 | 2.9 | 15.1 | 81.4 | 0 | 0 | 1.3 | 4.4 | 94.3 | 0 | 1.3 | 4.6 | 26.1 | 68.0 |
| 13 | N | 48 | 25 | 35 | 79 | 125 | 48 | 17 | 23 | 20 | 51 | 0 | 8 | 12 | 59 | 74 |
|  | % | 15.4 | 8.0 | 11.2 | 25.3 | 40.1 | 30.1 | 10.7 | 14.5 | 12.6 | 32.1 | 0 | 5.2 | 7.8 | 38.6 | 48.4 |
| 14 | N | 1 | 5 | 8 | 59 | 239 | 0 | 2 | 1 | 7 | 149 | 1 | 3 | 7 | 52 | 90 |
|  | % | .3 | 1.6 | 2.6 | 18.9 | 76.6 | 0 | 1.3 | .6 | 4.4 | 93.7 | .7 | 2.0 | 4.6 | 34.0 | 58.8 |
| 15 | N | 0 | 8 | 10 | 78 | 216 | 0 | 3 | 3 | 22 | 131 | 0 | 5 | 7 | 56 | 85 |
|  | % | 0 | 2.6 | 3.2 | 25.0 | 69.2 | 0 | 1.9 | 1.9 | 13.8 | 82.4 | 0 | 3.3 | 4.6 | 36.6 | 55.6 |
| 16 | N | 1 | 5 | 9 | 52 | 245 | 1 | 2 | 5 | 10 | 141 | 0 | 3 | 4 | 42 | 104 |
|  | % | .3 | 1.6 | 2.9 | 16.7 | 78.5 | .6 | 1.3 | 3.1 | 6.3 | 88.7 | 0 | 2.0 | 2.6 | 27.5 | 68.0 |
| 17 | N | 0 | 2 | 3 | 36 | 271 | 0 | 1 | 0 | 3 | 155 | 0 | 1 | 3 | 33 | 116 |
|  | % | 0 | .6 | 1.0 | 11.5 | 86.9 | 0 | .6 | 0 | 1.9 | 97.5 | 0 | .7 | 2.0 | 21.6 | 75.8 |
| 18 | N | 2 | 0 | 4 | 54 | 252 | 2 | 0 | 3 | 8 | 146 | 0 | 0 | 1 | 46 | 106 |
|  | % | .6 | 0 | 1.3 | 17.3 | 80.8 | 1.3 | 0 | 1.9 | 5.0 | 91.8 | 0 | 0 | .7 | 30.1 | 69.3 |
| 19 | N | 5 | 9 | 5 | 57 | 236 | 5 | 7 | 1 | 18 | 128 | 0 | 2 | 4 | 39 | 108 |
|  | % | 1.6 | 2.9 | 1.6 | 18.3 | 75.6 | 3.1 | 4.4 | .6 | 11.3 | 80.5 | 0 | 1.3 | 2.6 | 25.5 | 70.6 |
| 20 | N | 7 | 12 | 20 | 39 | 234 | 7 | 4 | 3 | 6 | 139 | 0 | 8 | 17 | 33 | 95 |
|  | % | 2.2 | 3.8 | 6.4 | 12.5 | 75.0 | 4.4 | 2.5 | 1.9 | 3.8 | 87.4 | 0 | 5.2 | 11.1 | 21.6 | 62.1 |
| 21 | N | 37 | 13 | 18 | 69 | 175 | 34 | 9 | 5 | 30 | 81 | 3 | 4 | 13 | 39 | 94 |
|  | % | 11.9 | 4.2 | 5.8 | 22.1 | 56.1 | 21.4 | 5.7 | 3.1 | 18.9 | 50.9 | 2.0 | 2.6 | 8.5 | 25.5 | 61.4 |
| 22 | N | 32 | 19 | 17 | 88 | 156 | 27 | 15 | 4 | 41 | 72 | 5 | 4 | 13 | 47 | 84 |
|  | % | 10.3 | 6.1 | 5.4 | 28.2 | 50.0 | 17.0 | 9.4 | 2.5 | 25.8 | 45.3 | 3.3 | 2.6 | 8.5 | 30.7 | 54.9 |
| 23 | N | 36 | 15 | 16 | 81 | 164 | 32 | 7 | 4 | 41 | 75 | 4 | 8 | 12 | 40 | 89 |
|  | % | 11.5 | 4.8 | 5.1 | 26.0 | 52.6 | 20.1 | 4.4 | 2.5 | 25.8 | 47.2 | 2.6 | 5.2 | 7.8 | 26.1 | 58.2 |
| 24 | N | 37 | 40 | 19 | 79 | 137 | 32 | 26 | 4 | 38 | 59 | 5 | 14 | 15 | 41 | 78 |
|  | % | 11.9 | 12.8 | 6.1 | 25.3 | 43.9 | 20.1 | 16.4 | 2.5 | 23.9 | 37.1 | 3.3 | 9.2 | 9.8 | 26.8 | 51.0 |
| 25 | N | 38 | 39 | 28 | 84 | 123 | 34 | 26 | 6 | 43 | 50 | 4 | 13 | 22 | 41 | 73 |
|  | % | 12.2 | 12.5 | 9.0 | 26.9 | 39.4 | 21.4 | 16.4 | 3.8 | 27.0 | 31.4 | 2.6 | 8.5 | 14.4 | 26.8 | 47.7 |
| 26 | N | 1 | 3 | 6 | 60 | 242 | 0 | 1 | 1 | 12 | 145 | 1 | 2 | 5 | 48 | 97 |
|  | % | .3 | 1.0 | 1.9 | 19.2 | 77.6 | 0 | .6 | .6 | 7.5 | 91.2 | .7 | 1.3 | 3.3 | 31.4 | 63.4 |
| 27 | N | 0 | 1 | 3 | 37 | 271 | 0 | 0 | 0 | 3 | 156 | 0 | 1 | 3 | 34 | 115 |
|  | % | 0 | .3 | 1.0 | 11.9 | 86.9 | 0 | 0 | 0 | 1.9 | 98.1 | 0 | .7 | 2.0 | 22.2 | 75.2 |
| 28 | N | 1 | 2 | 11 | 53 | 245 | 0 | 0 | 2 | 9 | 148 | 1 | 2 | 9 | 44 | 97 |
|  | % | .3 | .6 | 3.5 | 17.0 | 78.5 | 0 | 0 | 1.3 | 5.7 | 93.1 | .7 | 1.3 | 5.9 | 28.8 | 63.4 |
| 29 | N | 0 | 0 | 3 | 36 | 273 | 0 | 0 | 0 | 4 | 155 | 0 | 0 | 3 | 32 | 118 |
|  | % | 0 | 0 | 1.0 | 11.5 | 87.5 | 0 | 0 | 0 | 2.5 | 97.5 | 0 | 0 | 2.0 | 20.9 | 77.1 |
| 30 | N | 1 | 6 | 7 | 42 | 256 | 0 | 1 | 0 | 4 | 154 | 1 | 5 | 7 | 38 | 102 |
|  | % | .3 | 1.9 | 2.2 | 13.5 | 82.1 | 0 | .6 | 0 | 2.5 | 96.9 | .7 | 3.3 | 4.6 | 24.8 | 66.7 |
| 31 | N | 0 | 3 | 2 | 78 | 229 | 0 | 1 | 1 | 40 | 117 | 0 | 2 | 1 | 38 | 112 |
|  | % | 0 | 1.0 | .6 | 25.0 | 73.4 | 0 | .6 | .6 | 25.2 | 73.6 | 0 | 1.3 | .7 | 24.8 | 73.2 |
| 32 | N | 0 | 5 | 8 | 22 | 277 | 0 | 0 | 2 | 10 | 147 | 0 | 5 | 6 | 12 | 130 |
|  | % | 0 | 1.6 | 2.6 | 7.1 | 88.8 | 0 | 0 | 1.3 | 6.3 | 92.5 | 0 | 3.3 | 3.9 | 7.8 | 85.0 |

*The label for each item is presented in Table 3. Prosthodontic= Participants received long prosthodontic procedures, Conservative= Participants received short conservative dental procedures. SD: strongly disagree, D: disagree, Neu: neutral, A: agree, SA: strongly agree
